# The Fungicidal Activity of Thymol against *Fusarium graminearum* via Inducing Lipid Peroxidation and Disrupting Ergosterol Biosynthesis

**DOI:** 10.3390/molecules21060770

**Published:** 2016-06-18

**Authors:** Tao Gao, Hao Zhou, Wei Zhou, Liangbin Hu, Jian Chen, Zhiqi Shi

**Affiliations:** 1 Institute of Food Quality and Safety, Jiangsu Academy of Agricultural Sciences, 50 Zhongling Street, Nanjing 210014, China; 18551694895@163.com; 2 Key Lab of Food Quality and Safety of Jiangsu Province-State Key Laboratory Breeding Base, Jiangsu Provincial Department of agriculture and Forestry, 50 Zhongling Street, Nanjing 210014, China; 3 Key Laboratory of Control Technology and Standard for Agro-product Safety and Quality, Ministry of Agriculture, 50 Zhongling Street, Nanjing 210014, China; 4 Central Laboratory, Jiangsu Academy of Agricultural Science, 50 Zhongling Street, Nanjing 210014, China; m15751868639@163.com; 5 Department of Food Science, Henan Institute of Science and Technology, Xinxiang 453003, China; zhouweihistfood@163.com (W.Z.); hulb973@163.com (L.H.)

**Keywords:** thymol, *Fusarium graminearum*, lipid peroxidation, ergosterol biosynthesis

## Abstract

Thymol is a natural plant-derived compound that has been widely used in pharmaceutical and food preservation applications. However, the antifungal mechanism for thymol against phytopathogens remains unclear. In this study, we identified the antifungal action of thymol against *Fusarium graminearum*, an economically important phytopathogen showing severe resistance to traditional chemical fungicides. The sensitivity of thymol on different *F. graminearum* isolates was screened. The hyphal growth, as well as conidial production and germination, were quantified under thymol treatment. Histochemical, microscopic, and biochemical approaches were applied to investigate thymol-induced cell membrane damage. The average EC_50_ value of thymol for 59 *F. graminearum* isolates was 26.3 μg·mL^−1^. Thymol strongly inhibited conidial production and hyphal growth. Thymol-induced cell membrane damage was indicated by propidium iodide (PI) staining, morphological observation, relative conductivity, and glycerol measurement. Thymol induced a significant increase in malondialdehyde (MDA) concentration and a remarkable decrease in ergosterol content. Taken together, thymol showed potential antifungal activity against *F. graminearum* due to the cell membrane damage originating from lipid peroxidation and the disturbance of ergosterol biosynthesis. These results not only shed new light on the antifungal mechanism of thymol, but also imply a promising alternative for the control of Fusarium head blight (FHB) disease caused by *F. graminearum*.

## 1. Introduction

The filamentous ascomycete *Fusarium graminearum* Schwabe (teleomorph: *Gibberella zeae*) is a ubiquitous plant pathogen causing Fusarium head blight (FHB) in cereals. FHB has been considered one of the major threats to crop production worldwide because it results in yield losses as well as mycotoxin contamination in grains [1]. In China, FHB is an economically important disease, which has spread wildly from frequently occurring areas in eastern costal regions to the emerging outbreak area in northern and western wheat-growing regions [2,3,4]. The breeding of wheat varieties with resistance to FHB would seem to be the most promising approach to control this disease, but highly-resistant cultivars have not yet been developed for commercialization [5]. Consequently, FHB has been mainly controlled with chemical fungicides [6]. Benzimidazole (e.g., carbendazim) and triazole (e.g., tebuconazole) fungicides have been extensively applied to control FHB in China for many years [7,8]. However, *F. graminearum* populations can easily develop resistance to these chemical fungicides [8,9,10], even the recently developed fungicide phenamacril [11]. In addition, chemical fungicide residues with high toxicity pose a potential risk to the environment and human health. Therefore, it is essential to screen novel fungicides with low toxicity for the efficient control of FHB.

Thymol (5-methyl-2-(1-methylethyl) phenol, PubChem CID: 6989) is a natural monoterpene phenol found primarily in thyme, oregano, and tangerine peel. The antimicrobial activity of thymol against pathogenic microorganisms has been extensively reported in pharmaceutical research [12]. Thymol has been shown to possesses an anti-inflammatory property both *in vivo* and *in vitro* [13]. In addition, thymol has been listed by the U.S. Food and Drug Administration (FDA) as a food additive because it is considered a Generally Recognized as Safe (GRAS) substance [14]. As a preservative, thymol can help maintain quality and reduce the decay of fruits and vegetables by inhibiting microbial growth during postharvest storage [15,16,17]. The antimicrobial effect of thymol has been mainly linked to the reduction of ergosterol, resulting in the disruption of cell membrane integrity of microorganisms [18,19,20,21]. The U.S. Environmental Protection Agency (EPA) Office of Pesticide Programs determined that thymol has minimal potential toxicity and poses minimal risk [22], implying that thymol may have the potential to be developed as a novel botanical pesticide. The recent study suggests that an essential oils mixture from thyme and oregano can inhibit the growth of *Pseudomonas* species isolated from soybean leaves [23], but the antimicrobial activity of thymol against agricultural phytopathogens is poorly understood.

In this work, we identified the inhibitory effect of thymol on the growth of *F. graminearum in vitro*. A total of 59 *F. graminearum* isolates were tested to establish the baseline sensitivity to thymol. Thymol-induced disruption of cell membrane in *F. graminearum* was studied by physiological detection and microscopic observation. To get deeper insights into the effect of thymol on cell membrane damage, the involvement of lipid peroxidation and ergosterol biosynthesis was investigated. All of these results are important to help our understanding of the mode of action of thymol against *F. graminearum*, which will assist us in providing new reference data for the management of FHB caused by *F. graminearum*.

## 2. Results

### 2.1. Thymol Significantly Inhibited the Growth of F. graminearum

**Figure 1:**
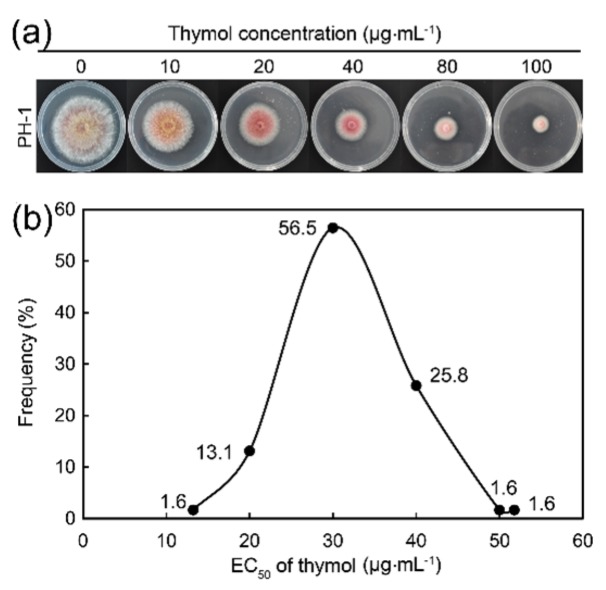
Effect of thymol on (**a**) standard strain PH-1 which is the model organism of *F. graminearum* and (**b**) other isolates of *F. graminearum*. (**a**) Strain PH-1 was cultivated on PDA plates containing thymol (0–100 μg·mL^−1^) for 3 days for photographing and the measurement of the diameter of colonies; (**b**) Distribution of thymol EC_50_ values for 59 *F. graminearum* isolates. A total of 59 *F. graminearum* isolates were cultured on PDA plates containing thymol (0–100 μg·mL^−1^) for 3 days for the measurement of the diameter of colonies. Thymol EC_50_ value for each isolate was calculated for the establishment of a baseline sensitivity curve. Each result was the mean of three replicates.

First, we determined the effect of thymol on *F. graminearum* standard strain PH-1 which is the model organism of *F. graminearum*. Thymol showed strong inhibition on hyphal growth of PH-1 with the EC_50_ value at 28.68 μg·mL^−1^ (Figure 1a). Then, we screened the thymol sensitivity of 59 *F. graminearum* isolates collected from Jiangsu province in 2014. The EC_50_ values of thymol for these isolates ranged from 22.53 to 51.76 μg·mL^−1^, with a mean value of 26.3 μg·mL^−1^ (Figure 1b). These results indicated that thymol had a severe adverse effect on the growth of *F. graminearum*. In order to study the biochemical mechanism for the antifungal activity of thymol, we selected *F. graminearum* standard strain PH-1 in the following experiments.

### 2.2. Thymol Affected Conidia Production and Conidia Germination of F. graminearum

**Figure 2:**
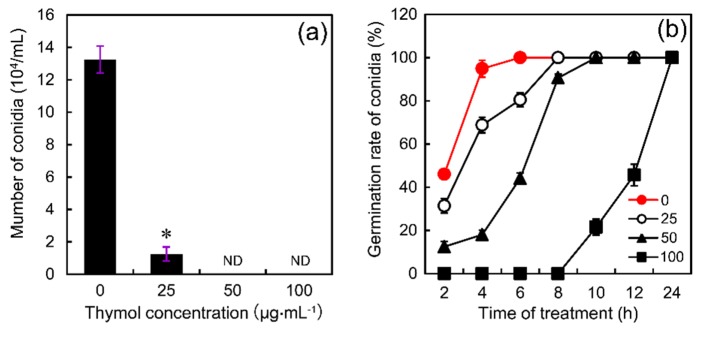
Effect of thymol on (**a**) conidia production and (**b**) conidia germination of *F. graminearum* (PH-1). (**a**) Conidia were induced in MBB medium containing thymol (0, 25, 50, and 100 μg·mL^−1^) for 5 days. Then the conidia were counted under microscopy. (**b**) The produced conidia were cultured in YEPD medium containing thymol (0, 25, 50, and 100 μg·mL^−1^) up to 24 h. The germinated conidia were monitored for the calculation of germination rate. Vertical bars represent standard deviations of the mean (*n* = 3). Asterisk indicates that mean values are significantly different (*p* < 0.05) between the treatment and the control. ND: not detected.

To investigate the effect of thymol on the production of conidia, thymol at the final concentration of 25–100 μg·mL^−1^ was added to MBB medium before the formation of conidia. Compared to the control, the number of conidia significantly decreased by 91% at 25 μg·mL^−1^ of thymol level. Thymol at 50–100 μg·mL^−1^ completely inhibited the production of conidia (Figure 2a). To test the effect of thymol on conidia germination, thymol at the final concentration of 25–100 μg·mL^−1^ was added to YEPD medium containing the produced conidia. Then the germination rate of conidia was monitored in a time-course experiment. Basically, thymol did not impact the final germination rate of conidia, but it could delay the process of conidia germination. In the control group, all the conidia could germinate completely after 6 h. However, it took 8, 10, and 24 h to achieve 100% germination rate at 25, 50, and 100 μg·mL^−1^ of thymol levels, respectively (Figure 2b). These results suggested that thymol could remarkably affect the conidial development of *F. graminearum*.

### 2.3. Thymol Changed the Morphology of F. graminearum

**Figure 3:**
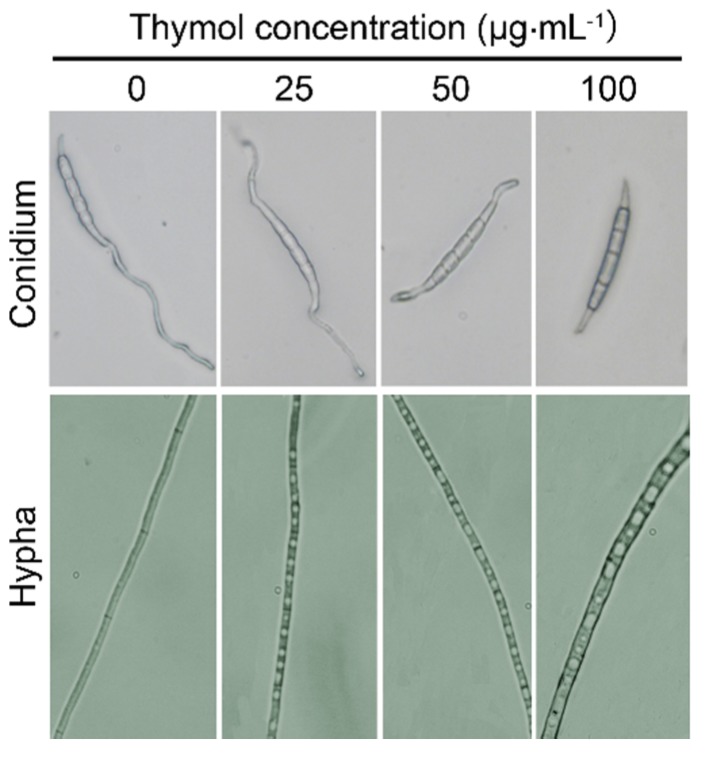
Effect of thymol on the morphology of conidium and hypha of *F. graminearum* (PH-1). The fresh conidia were treated with thymol at 0, 25, 50, and 100 μg·mL^−1^ for 24 h, respectively. Then the samples were visualized and photographed under light microscope.

**Figure 4:**
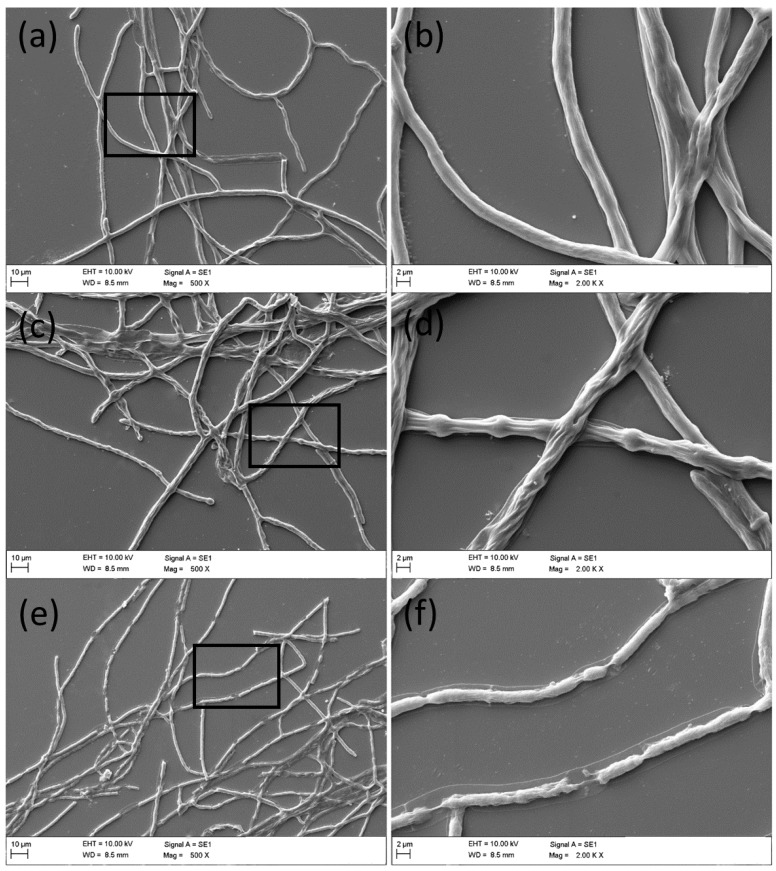
SEM observation of the hyphal morphology of *F. graminearum* (PH-1) under thymol treatment. Hyphae were treated with thymol at (**a**,**b**) 0; (**c**,**d**) 25; and (**e**,**f**) 100 μg·mL^−1^ for 24 h. The micrographs were taken at the magnification of (**a**,**c**,**e**) 500× and (**b**,**d**,**f**) 2000×, respectively.

Light microscopy was used to observe the morphology of conidia and hyphae under thymol treatment. After treatment for 12 h, the germination of conidia could be prohibited, but the morphology of the conidium itself was not changed significantly (Figure 3). However, thymol-treated hyphae with increased vacuoles inside of cells became bolder and shorter as compared to the control (Figure 3). Furthermore, the mycelial growth of *F. graminearum* under SEM clearly revealed morphological alterations in hyphae under thymol treatment (Figure 4). Compared to the control group with regular and smooth hyphae (Figure 4a,b), treatment with 25 μg·mL^−1^ of thymol resulted in a loss of cell shape and the irregular shrinkage of hyphae (Figure 4c,d). Treatment with thymol at high concentration (100 μg·mL^−1^) led to significant collapse and breakdown of hyphae (Figure 4e,f).

### 2.4. Thymol Induced Cell Membrane Injury of F. graminearum

**Figure 5:**
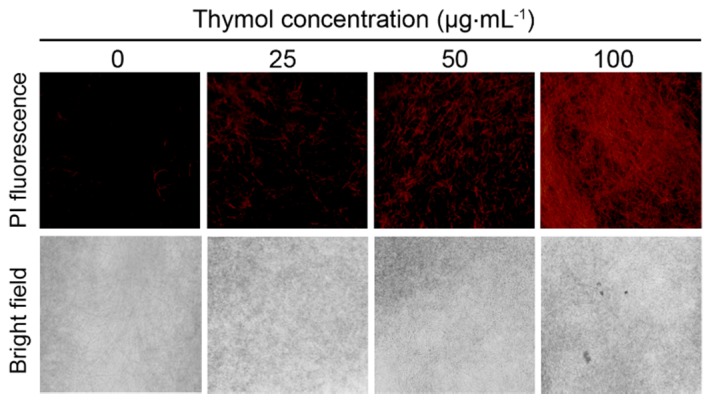
PI staining observation of the hyphae of *F. graminearum* (PH-1) under thymol treatment. Hyphae treated with thymol at 0, 25, 50, and 100 μg·mL^−1^ for 24 h were incubated in PI solution for 20 min. Then, the photos were captured under fluorescent microscope.

To test the effect of thymol on the cell membrane of *F. graminearum*, we performed propidium iodide (PI) staining that has been frequently used to indicate cell membrane damage [24]. The PI-stained hyphae of *F. graminearum* showed more extensive red fluorescence in the presence of thymol than that of control (Figure 5), indicating that thymol treatment let to cell membrane injury.

**Figure 6:**
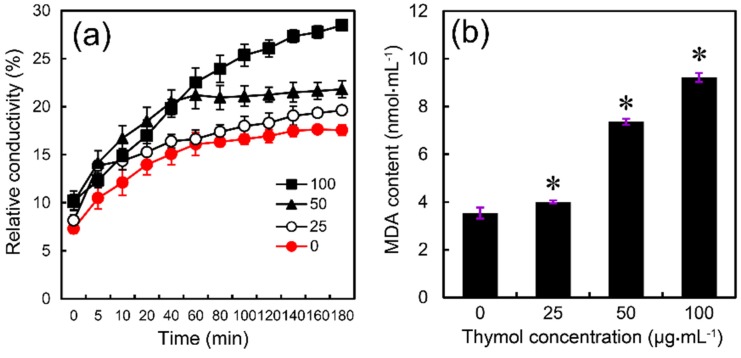
Effect of thymol on (**a**) the relative conductivity and (**b**) MDA content of the mycelia of *F. graminearum* (PH-1). Mycelia treated with thymol at 0, 25, 50, and 100 μg·mL^−1^ for 24 h were harvested for the measurements. Each value is presented as the average of three replicates. Asterisk indicates that mean values are significantly different (*p* < 0.05) between the treatment and the control.

Treatment with thymol resulted in a remarkable increase in the relative conductivity of *F. graminearum* as compared to the control (Figure 6a), suggesting that thymol could induce electrolyte leakage by enhancing cell membrane permeability in *F. graminearum*. Malondialdehyde (MDA) concentration has been frequently used as an index of lipid peroxidation, indicating the oxidative injury of the cell plasma membrane [25]. Compared to the control, the MDA content significantly increased by 13%, 108%, and 160% at 25, 50, and 100 μg·mL^−1^ of thymol levels, respectively (Figure 6b). These results suggested that thymol treatment induces cell membrane damage in *F. graminearum*.

**Figure 7:**
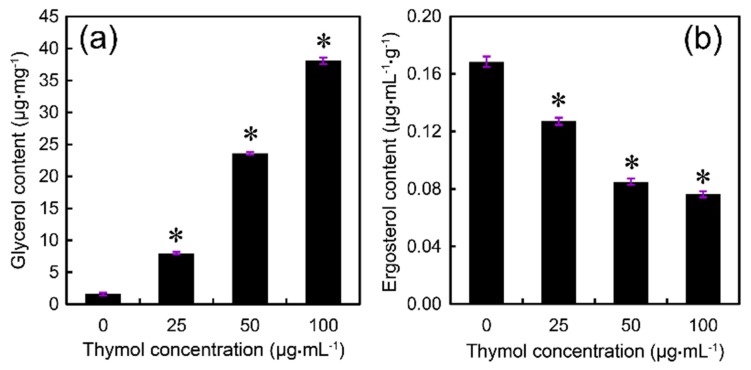
Effect of thymol on (**a**) glycerol and (**b**) ergosterol content of mycelia of *F. graminearum* (PH-1). Mycelia treated with thymol at 0, 25, 50, and 100 μg·mL^−1^ for 24 h were harvested for the measurements. Each value was presented as the average of three replicates. Asterisk indicates that mean values are significantly different (*p* < 0.05) between the treatment and the control.

Electrolyte leakage resulting from cell membrane damage can trigger osmotic stress responses in fungi. It has been shown that intracellular glycerol plays an important role in the response of fungi to osmotic stress [26]. In the present study, we found that the glycerol content increased significantly in thymol-treated *F. graminearum* in a dose-dependent manner (Figure 7a), suggesting that glycerol accumulation could be induced by thymol in order to maintain osmotic balance. Ergosterol—the major sterol component of fungal cell membrane—is important for maintaining membrane integrity and cell function [27]. Compared to the control, treatment with thymol at 25, 50, and 100 μg·mL^−1^ resulted in a decrease in ergosterol content in *F. graminearum* by 25%, 50%, and 55%, respectively (Figure 7b).

**Figure 8:**
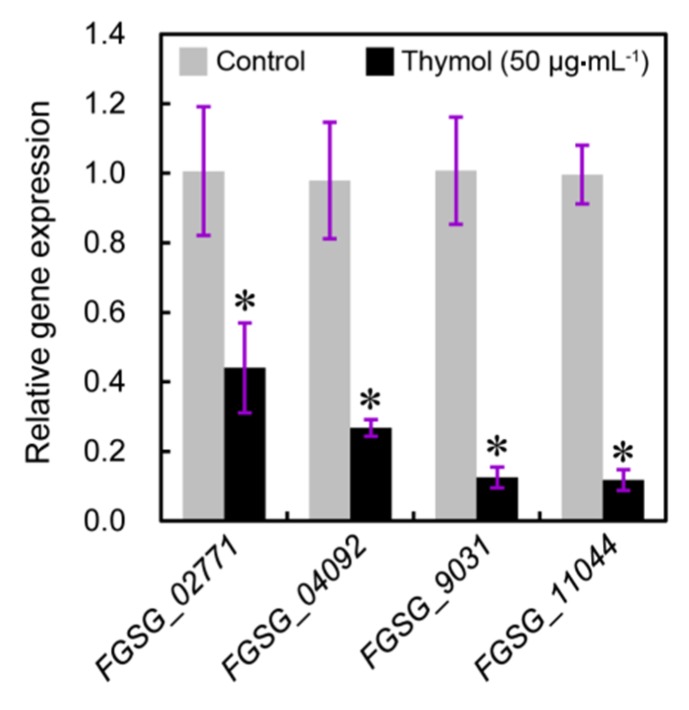
Effect of thymol on the expression of genes related to ergosterol biosynthesis in *F. graminearum* (PH-1). Mycelia treated with thymol at 0 and 50 μg·mL^−1^ for 24 h were harvested for RNA extraction and quantitative reverse transcription-polymerase chain reaction (qRT-PCR) analysis. Each value was presented as the average of three replicates. Asterisk indicates that mean values are significantly different (*p* < 0.05) between the treatment and the control.

To test the effect of thymol treatment on the biosynthetic pathway of ergosterol in *F. graminearum*, we detected the expression of several genes related to ergosterol biosynthesis. *FGSG_04092* indicates *cyp51A* encoding the cytochrome P450 sterol 14α-demethylase [28]. *FGSG_0277*1, *FGSG_09031*, and *FGSG_11044* are the homologues *KES1* that are indispensable for the biosynthesis and distribution of ergosterol [29,30]. The results from quantitative reverse transcription-polymerase chain reaction (qRT-PCR) suggested that treatment with thymol at 50 μg·mL^−1^ for 24 h resulted in a significant decrease in the expression of four tested genes as compared to the control groups (Figure 8).

## 3. Discussion

Essential oils originating from medicinal plants have always been of great interest to researchers because of their great potential to inhibit pathogens as well as their medicinal properties [31,32]. Recently, some essential oils have been proposed to inhibit the growth of phytopathogens [33]. The medicinal properties of thymol have been extensively reported, but its antimicrobial activities against phytopathogens—as well as the manipulating mechanism—are not well understood. The effective control of FHB disease caused by *F. graminearum* is a big challenge worldwide [34]. Here we proposed that both oxidative injury and the disruption of ergosterol biosynthesis contributed to thymol-induced cell membrane damage in *F. graminearum*.

The results from sensitivity testing suggested that thymol showed extensive antifungal activity against various *F. graminearum* isolates. Thymol showed a detrimental effect on both conidial production and hyphal growth of strain PH-1. Thymol was able to delay but not to affect the final germination of already-produced conidia in strain PH-1, which was consistent with the light microscopic observation of conidia. It has been reported that tannic acid and extracts from Chinese galls could inhibit the germination of conidia in local *F. graminearum* isolates from Switzerland [35]. This disparity implies the different action modes among different plant extracts against *F. graminearum*. Vacuoles in filamentous fungi play vital roles in response to osmotic stress [36]. In the present study, thymol induced considerable generation of vacuoles in hypha, suggesting that thymol might trigger an osmotic stress response in *F. graminearum*. This phenomenon could be verified by the accumulation of glycerol, which is a typical indicator of osmotic stress in fungi. It has been reported that fludioxonil-induced glycerol accumulation and osmotic stress in filamentous fungus *Neurospora crassa* via the activation of the high-osmolality glycerol (HOG) pathway mediated by mitogen-activated protein kinase (MAPK) cascade [37]. Notably, the anti-inflammatory effect of thymol has been closely associated with the regulation of MAPK signaling pathways in mammalian cells [13,38]. Therefore, whether and how thymol modulates MAPK signaling in *F. graminearum* would be an interesting topic to be further elucidated. 

The antibacterial activity of plant-derived phenols has been partly attributed to their hydrophobicity, rendering the membrane permeable and leading to leakage of cell contents [39]. In this work, the results obtained from PI staining suggested that thymol could induce cell membrane damage in *F. graminearum*, which further resulted in electrolyte leakage from cells. It has been reported that yeast cells treated with thymol at 64–500 μg·mL^−1^ could be stained extensively by PI as compared to the control [18,21]. In addition, Chauhan and Kang [19] have found that thymol at 750 μg·mL^−1^ could induce the disruption of cell membrane in *Salmonella* ser. *typhimurium*. The SEM results from their work showed the significant leakage of cellular materials and altered morphology of bacterial cells induced by thymol [19]. In our present study, thymol at 25–100 μg·mL^−1^ could induce morphological alteration in the hyphae of *F. graminearum* due to cell membrane damage. Thus, it is possible that thymol tends to destroy cell membrane in different kinds of microorganisms. Reactive oxygen species (ROS) originated from NADPH oxidase located in the plasma membrane is one of the major factors inducing the lipid peroxidation of eukaryotic cell membrane under various environmental stimuli [40,41]. In our recent study, the fungicidal action of thymol against spores of *Aspergillus flavus* has been closely linked to the over-generation of ROS originating from NADPH oxidase [42]. In addition, thymol modulates the phagocytotic activity of macrophage by enhancing NADPH oxidase-dependent generation of ROS [43]. In *F. graminearum*, whether thymol induced the lipid peroxidation of cell membrane through NADPH-dependent ROS generation remains to be investigated.

Fungal ergosterol is a classical drug target because it is important for plasma membrane structure and function and for localization of plasma membrane proteins [44,45]. Thymol-induced significant impairment of ergosterol biosynthesis has been found in *Candida* [18]. In the present study, we obtained a similar result, showing that thymol pronouncedly decreased the content of ergosterol in *F. graminearum*, which may result from the decrease in the expression of the genes related to ergosterol biosynthesis. *cyp51* has been reported as the effective target of azole fungicides [28,46]. The down-regulation of the expression of *cyp51A* induced by thymol suggests that thymol disrupts ergosterol biosynthesis in a manner similar to that of azole. In addition, *KES1* encoding for oxysterol-binding protein not only contributes to ergosterol biosynthesis but also regulates sterol-lipid distribution, endocytosis, and Golgi apparatus [29,30,47]. In the present study, thymol treatment induced significant repression of three *KES1* homologues in *F. graminearum*. Further studies may reveal the possibly novel fungicidal mechanism of thymol by targeting *KES1-*like genes.

Collectively, we demonstrated that the naturally plant-derived compound thymol exhibited strong inhibitory activity against *F. graminearum in vitro*. The antifungal effect of thymol may be attributed to its capability to cause cell membrane damage by inducing lipid peroxidation and inhibiting ergosterol biosynthesis. Despite the observation in this study, there are still many works required. Are there specific targets for thymol to provide possible explanations for thymol-induced disturbance of membrane homeostasis as well as ergosterol in *F. graminearum*? Are there any other manipulating mechanisms for the antifungal activity of thymol against *F. graminearum*? Is the development of thymol resistance in *F. graminearum* possible? The further understanding of these questions will accelerate the investigation of the fungicidal properties of thymol, which in turn will help the development of an alternative principle to control FHB caused by *F. graminearum* infection.

## 4. Materials and Methods

### 4.1. Media, Strains, and Chemicals

The strains were grown on Potato Dextrose Agar (PDA; 200 g of potato, 20 g of dextrose, 15 g of agar, and 1 L of distilled water) medium and Mung Bean Broth (MBB) medium for mycelial growth assay and sporulation assay, respectively [48]. The Yeast Extract Peptone Dextrose (YEPD) medium was used for conidial germination assay. From 2013 to 2014, a total of 59 isolates of *F. graminearum* were collected from infected wheat plants in different geographic wheat fields in Jiangsu Province of China. These isolates were generated from a single spore and maintained on PDA slants at 4 °C. The standard strain of *F. graminearum* PH-1 was used to investigate the detailed physiological and biochemical responses under thymol treatment. Thymol (a.i. 99%; Produced in Germany and repacked by Nanjing Hengxin chemical Co., Ltd. Nanjing, China) was dissolved in ethanol at 1 × 105 µg·mL^−1^ as stocking solution stored at 4 °C in darkness. Solutions were diluted with distilled water into different concentrations as required. The fluorescent probe propidium iodide (PI) was obtained from Beyotime Biotechnology Research Institute, China.

### 4.2. Determination of Baseline Sensitivity of F. graminearum to Thymol

The sensitivity of 59 isolates of *F. graminearum* to thymol was determined *in vitro* by transferring plugs (5 mm in diameter) of mycelium from the leading edge of an actively growing colony to a series of PDA plates containing 0, 10, 20, 40, 60, 80, and 100 μg·mL^−1^ of thymol, respectively. The diameters (minus the diameter of the inoculation plug) of the colonies were measured after incubation for 3 days at 25 °C in darkness. The growth inhibition as percent of control was calculated. The median effective thymol concentration (EC_50_) for the isolates was calculated based on linear regression of colony diameter on log-transformed fungicide concentration [49]. The bioassay data were obtained from the mean of three replicates.

### 4.3. Measurement of Conidiation Production, Conidiation Germination, and Mycelial Morphology of F. graminearum

For conidiation assay, ten mycelial plugs (5 mm in diameter) taken from the margin of a 3-day-old colony of each strain were added to a 250 mL flask containing 100 mL of MBB for shake culture, which contained 0, 25, 50, and 100 μg·mL^−1^ of thymol, respectively. The flasks were incubated at 25 °C for 5 days with shaking (175 rpm). The number of conidia in the MBB in each flask was determined with a haemacytometer and microscope [50]. The bioassay data were obtained from the mean of three replicates.

For the investigation of conidial germination, mycelial morphology, and mycelial plugs, the parental strain PH-1 was cultured in YEPD medium containing 0, 25, 50, or 100 μg·mL^−1^ of thymol for up to 24 h. Microscopic examination was carried out using germ tubes germinated from spores for 2, 4, 6, 8, 10, 12, and 24 h, respectively. Sample sections were prepared and visualized using a light microscope (Olympus IX-71, Tokyo, Japan).

### 4.4. Scanning Electron Microscopy (SEM)

Mycelial plugs (5 mm in diameter) of *F. graminearum* at the same age were taken from the control and both thymol treatments (25 and 100 μg·mL^−1^) for SEM observation. The samples were fixed in 2.5% glutaraldehyde for 2–4 h. Then the samples were transferred to alcohol with increasing concentrations (30%, 50%, and 70%) for 15 min followed by 20 min in 80%, 90%, 95%, and 100% of alcohol, consecutively. The graded aqueous series of acetone (25%, 50%, 75%, and 100%) was critical point dried with CO_2_ using acetone as an intermediate fluid. After that, the fungal samples were visualized and photographed by SEM (EVO-LS10, ZEISS, Jena, Germany).

### 4.5. Determination of Relative Conductivity

The relative conductivity of thymol-treated *F. graminearum* was measured according to the method described by Duan *et al.* [51]. Mycelial plugs (5 mm diameter) from the margins of 3-day-old colonies on PDA were placed in 250 mL flasks (five plugs per flask) containing 100 mL of PDB. The flasks were placed on a rotary shaker (175 rpm at 25 °C). After 24 h, partial flasks were treated with thymol at the ultimate concentration of 25, 50, or 100 μg·mL^−1^. After shaking for an additional 24 h, mycelia were collected on double gauze and washed twice with double-distilled water. After filtration in vacuum for 20 min, 0.5 g of mycelia per sample was suspended in 20 mL of double-distilled water. The electrical conductivity of the double-distilled water was measured with a conductivity meter (CON510 Eutech/Oakton, Singapore) at 0, 5, 10, 20, 40, 60, 80, 100, 120, 140, 160, and 180 min, respectively, to assess the extent of leaching of cell contents through cell membranes. After 180 min, the mycelia were boiled for 5 min for the measurement of final conductivity. The relative conductivity of mycelia was calculated as:

Relative conductivity (%) = Conductivity/Final conductivity × 100

### 4.6. Determination of Glycerol Content

Glycerol in mycelia was measured by the cupric glycerinate colorimetric method with some modification [52]. The PH-1 strain was grown in potato dextrose broth (PDB) for 24 h at 25 °C on a shaker. After treatment with 0, 25, 50, or 100 μg·mL^−1^ of thymol for 24 h, mycelia of each concentration were harvested and ground with liquid nitrogen. The mycelial powders were transferred to a 2 mL centrifuge tube containing 1 mL of sterile water. A glycerol detection kit (Beijing Applygen Technologies Inc., Beijing, China) was selected to determine the glycerol concentration based on the glycerol phosphate oxidase method and GPO Trinder enzymatic reactions.

### 4.7. Determination of Lipid Peroxidation

The concentration of malondialdehyde (MDA) was determined as an indicator of the level of lipid peroxidation in thymol-treated *F. graminearum*. A MDA detection kit (A003; Nanjing Jiancheng Bioengineering Institute, Nanjing, China) was selected to determine the MDA level based on the spectrophotometric determination of the reaction between MDA and 1,3-diethyl-2-thiobarbituric acid (TBA) assisted by trichloroacetic acid (TCA) [53].

### 4.8. Determination of Ergosterol Content

For extraction of ergosterol, 100 μL of the spore suspension (105 spores/mL) was incubated in 100 mL YEPD at 25 °C in a shaker (180 rpm) for 24 h. Then, thymol was added at the final concentration of 0, 25, 50, or 100 μg·mL^−1^. After shaking for 48 h, mycelia were collected on double gauze and washed twice with double-distilled water. The harvested mycelia were dried at 60 °C for 3 h. Total ergosterol was extracted from dried mycelia by using a previously published protocol [54]. Ergosterol concentrations were quantified with a high-performance liquid chromatography (HPLC) system, Prominence SPD-20A. Ergosterol was separated at room temperature on a hypersil BDS C18 250 nm × 4.6 nm, 5 μm analytical column using 100% methanol (chromatography pure) as mobile phase. The detection wavelength was 282 nm. The identification of ergosterol was based on retention time and co-chromatography of commercial standard of ergosterol (Sigma-Aldrich, St. Louis, MO, USA).

### 4.9. Histochemical Detection Cell Membrane Permeability

The cell membrane permeability was detected with a fluorescent probe (PI) that can only enter membrane-compromised cells to bind nucleic acids [24]. Fresh mycelia were treated with thymol at the ultimate concentration of 25, 50, or 100 μg·mL^−1^ for 24 h. Then, the mycelia were harvested and incubated in 2 μM of PI for 20 min, and rinsed with distilled water three times followed by the visualization (excitation 535 nm and emission 615 nm) by a fluorescence microscope (ECLIPSE, TE2000-S, Nikon Instruments Co., Ltd, Shanghai, China).

### 4.10. Analysis of Gene Expression

The real-time quantitative reverse transcription-polymerase chain reaction (qRT-PCR) was selected to quantify the expression levels of the genes related to ergosterol biosynthesis. The sequences of four tested genes (accession number: *FGSG_0277*1, *FGSG_04092*, *FGSG_09031*, and *FGSG_11044*) were retrieved from *F. graminearum* genome (http://www.broadinstitute.org/annotation/genome/fusarium_group/MultiHome.html) for the design of primers. Total RNA was isolated from mycelia using Trizol (Invitrogen, Waltham, MA, USA) according to the manufacturer’s instructions. The first-strand cDNA synthesized with the PrimeScript^®^ RT reagent kit (TaKaRa, Kusatsu, Japan) was used as template for qRT-PCR analysis (Applied Biosystems 7500 Fast Real-Time PCR System, LifeTechnologies™, Carlsbad, CA, USA). The primers used for amplifying the target genes are as follows: *FGSG_0277*1, forward 5′-CAGGCAAGGAGAAGGATGTT-3′ and reverse 5′-GGGTAGACTTGGAGTTGGATTT-3′; FGSG_04092, forward 5′-CCAAGGCAATGGCTGAGATA-3′ and reverse 5′-TGTTCGAATGCCCCCTTTT-3′; *FGSG_09031*, forward 5′-GCATCTGCAGCGAAACATAC-3′ and reverse 5′-TCCTCTCCCGGAACACTATTA-3′; *FGSG_11044*, forward 5′-CGGAGGAGCTTCTAGGTATGA-3′ and reverse 5′-CCGCTCCATCAACTCCAATAA-3′. To standardize the results, the relative abundance of elongation factor 1-α (EF1-α, FGSG_08811, forward 5′-CTTACTGCCTCCACCAACTG-3′ and reverse 5′-TGACGTTGGAAGGAGCGAAG-3′) was also determined and used as the internal standard.

### 4.11. Data Analysis

The effective concentration at which mycelial growth was inhibited by 50% (EC_50_) value for each isolate was estimated based on linear regression of the log of the colony diameter *versus* the thymol concentration. Each result was presented as the mean ± standard deviation (SD) of at least three replicated measurements. The significant differences between treatments were statistically evaluated by SD and one-way analysis of variance (ANOVA) using SPSS 14.0 (Statistical Package for the Social Science, SPSS Inc., Chicago, IL, USA). The data between two different treatments were compared statistically by ANOVA, followed by F-test if the ANOVA result was significant at *p* < 0.05.
